# Identification of a Geranylgeranyl reductase gene for chlorophyll synthesis in rice

**DOI:** 10.1186/2193-1801-3-201

**Published:** 2014-04-24

**Authors:** Pingyu Wang, Chunmei Li, Yang Wang, Rui Huang, Changhui Sun, Zhengjun Xu, Jianqing Zhu, Xiaoling Gao, Xiaojian Deng, Pingrong Wang

**Affiliations:** Rice Research Institute, Sichuan Agricultural University, Chengdu, 611130 Sichuan, China

**Keywords:** Geranylgeranyl reductase, *Chl P* gene, Chlorophyll, Rice

## Abstract

Geranylgeranyl reductase (CHL P) catalyzes the reduction of geranylgeranyl diphosphate to phytyl diphosphate, and provides phytol for both Chlorophyll (Chl) and tocopherol synthesis. In this study, we isolated a yellow-green leaf mutant, *502ys*, in rice (*Oryza sativa*). The mutant exhibited reduced level of Chls, arrested development of chloroplasts, and retarded growth rate. The phenotype of the *502ys* mutant was controlled by by a recessive mutation in a nuclear gene on the long arm of rice chromosome 2. Map-based cloning of the mutant resulted in the identification of an *OsChl P* gene (*LOC_Os02g51080*). In the *502ys* mutant, a single base pair mutation was detected at residue 1279 in DNA sequence of the gene, resulting in an amino acid change (Gly-206 to Ser) in the encoded protein. HPLC analysis of Chls indicated that the majority of Chl molecules are conjugated with an unsaturated geranylgeraniol side chain, in addition to small amount of normal Chls in the mutant. Furthermore, the mutant phenotype was complemented by transformation with the wild-type gene. Therefore, this study has confirmed the *502ys* mutant resulted from a single base pair mutation in *OsChl P* gene.

**Electronic supplementary material:**

The online version of this article (doi:10.1186/2193-1801-3-201) contains supplementary material, which is available to authorized users.

## Introduction

Chlorophyll (Chl) is the main component of the photosynthetic pigments. Chl molecules consist of two parts: a hydrophilic tetrapyrrole moiety (chlorophyllide, Chlide) and a hydrophobic alcohol moiety (phytyl, Phy), which are formed from the precursor molecules glutamyl-tRNA and isopentenyl diphosphate, respectively, in two different pathways of tetrapyrrole and isoprenoid biosynthesis. The tetrapyrrole biosynthesis, entirely locating in plastids, has been extensively studied with various organisms by biochemical and genetic methods. So far, almost all genes for 15 steps of tetrapyrrole biosynthetic pathway have been identified in higher plants represented by Arabidopsis (Beale 2005;Nagata et al. 2005;Wu et al. 2007;Islam et al. 2008;Wang et al. 2010,2013;Sakuraba et al. 2013).

The branched isoprenoid pathway is rather complex and comprises enzymatic steps in at least two compartments. In plants, Phy represents the side chain of Chls, tocopherols (TP) and phylloquinones, and is necessary for their integration into plastidmembranes (Soll et al. 1980,1983;Soll 1987;Bollivar et al. 1994). In both Chl and TP synthesis, the Phy chain is provided by geranylgeranyl pyrophosphate (GGPP), a plastidial isoprenoid, formed by four molecules of isopentenyl pyrophosphate (IPP), which are derived from the cytosolic and chloroplastidic pathways (Rohmer et al. 1993;Lichtentaler et al. 1997). In Chl synthesis, GGPP can either be reduced to phytyl pyrophosphate (PhyPP) and esterified with Chlide to generate phytyl Chl (Chl_Phy_), or first esterified with Chlide to form geranylgeranylated Chl (Chl_GG_) and then stepwise reduced into Chl_Phy_ (Soll et al. 1983;Bollivar et al. 1994;Keller et al. 1998;Chew et al. 2008). In the TP pathway, tocopherols are generally believed to arise from the condensation of homogentisic acid and PhyPP (Schultz et al. 1985;Soll et al. 1980;Collakova and DellaPenna 2001;Savidge et al. 2002;Cahoon et al. 2003). Therefore, CHL P is demonstrated to provide Phy for both Chl and TP synthesis.

CHL P catalyzes the reduction of Chl_GG_*a* into Chl_Phy_*a* as well as the reduction of free GGPP into PhyPP (Keller et al. 1998). Genes encoding CHL P have been characterized in photosynthetic bacteria such as *Syneclrocysti*s sp. PCC6803, *Rhodobacter sphaeroides* and *Chlorobaculum tepidum* (Addlesee et al. 1996;Addlesee and Hunter. 1999;Chew et al. 2008), and in higher plants such as Arabidopsis (*Arabidopsis thaliana*), tobacco (*Nicotiana tabacum*), peach (*Prunus persica*), olive (*Olea europaea*) and sesame (*Sesamum indicum*) (Keller et al. 1998;Tanaka et al. 1999;Giannino et al. 2004;Bruno et al. 2009;Park et al. 2010). But until recently, no *Chl P* gene was identified in monocotyledonous plants (Zhou et al. 2013).

In this study, we isolated a rice yellow-green leaf mutant, *502ys*, from ethyl methanesulfonate (EMS) mutagenesis. The mutant exhibited reduced level of Chls, arrested development of chloroplasts, and retarded growth rate. Map-based cloning of the mutant resulted in identification of the *OsChl P* gene. In the *502ys* mutant, a single base pair mutation was detected in DNA sequence of the gene, resulting in an amino acid change in the encoded protein. HPLC analysis of Chls indicated that the majority of Chls molecules are conjugated with an unsaturated geranylgeraniol side chain, in addition to small amount of normal Chls in the mutant. Furthermore, the mutant phenotype was complemented by transformation with the wild-type gene. Therefore, this study has confirmed the *502ys* mutant resulted from a single base pair mutation in *OsChl P* gene.

## Materials and methods

### Plant materials and mapping population

The *502ys* mutant was a yellow-green leaf mutant isolated from the progeny of *japonica* cv Nipponbare treated with 0.7% of ethyl methanesulfonate (v: v, Sigma). The F_2_ mapping population was generated by crossing the *502ys* mutant with normal green *indica* cv Minghui 63. All rice materials were planted under natural conditions in April to August, in Wenjiang District (Latitude 30°42′N, Longitude 103°50′E, and Altitude 539.3 m), Chengdu City, Sichuan, China.

### Marker development

The SSR markers were obtained from Gramene (http://archive.gramene.org/markers/microsat/) based on the SSR linkage map constructed by McCouch et al. (2002). To narrow down the region of the target locus, the BLAST search in the National Center for Biotechnology Information database was conducted to find insertion/deletion (InDel) sequence divergence between *japonica* cv Nipponbare and *indica* cv 9311 around this locus (http://www.ncbi.nlm.nih.gov/BLAST/), and InDel markers were designed around the sequence divergence including 15 to 100 bp InDel using the Primer 5.0 software.

### Sequence analysis

The full-length DNA and protein sequences of *OsChl P* and its homologs were retrieved from GenBank (http://www.ncbi.nlm.nih.gov). The chloroplast signal peptide was predicted with http://www.cbs.dtu.dk/services/TargetP (Emanuelsson et al. 2000). Multiple sequence alignment and phylogenetic analysis were conducted using DNAMAN version 6.0 (Lynnon Biosoft).

### Transmission electron microscopy analysis

Rice leaf samples of *502ys* and Nipponbare were harvested from four-week-old seedlings grown in natural conditions. Leaf sections were fixed in a solution of 3% glutaraldehyde and post-fixed with 1% osmium tetroxide. The tissues were dehydrated in a gradient acetone series and embedded in Epon812 medium prior to thin sectioning. The samples were stained with uranyl acetate and Reynolds’ lead citrate, and observed under a transmission electron microscope (Hitachi H-600IV, Japan).

### Analysis of pigments

Chls were extracted from 0.2 g fresh leaves of the *502ys* mutant and its wild-type Nipponbare with 80% acetone, and Chl contents were determined with UV-1700 UV-visible spectrophotometer (Shimadzu) according to the method of Arnon (1949).

Chls used for HPLC analysis were extracted from fresh rice leaf tissue of four-week-old *502ys* and wild-type plants with 100% acetone. The extract was centrifuged at 7,197 g (Eppendorf 5430R; 7,830 rpm) for 15 min, and the supernatants were subjected to HPLC on a C18 column (Eclipse XDB-C18, 4.6 mm i.d. × 150 mm long, 5 μm; Agilent) and eluted with solvent (methanol: acetonitrile: acetone = 1:3:1) at a flow-rate of 1.0 mL min^−1^ at 40°C, as previously described (Zapata et al. 2000;Nakanishi et al. 2005;Tanaka et al. 1999,2010;Zhou et al. 2013). Elution profiles were monitored by measuring A_660_, and Chl *a* and *b* standards (Sigma) were used as control.

### Complementation of the *502ys* mutant

For complementation of the *502ys* mutation, the full-length 2,141-bp *OsChl P* (*LOC_Os02g51080*) gene was amplified using primer 1 F (5′-ACA*TCTAGA*ATGACCTCGCTGTCGTCC-3′) and primer 1R (5′-AGC*CTGCAG*TCACAAGGTGACCTTCTC-3′) from the wild-type Nipponbare. The primers incorporated an *Xba*I site at the N-terminal end and a *Pst*I site at the C-terminal end of the gene. The PCR products were inserted into the pMD19-T vector (TaKaRa) and sequenced to obtain the correct clone pMD-*OsChl P*. The pMD-*OsChl P* plasmid was then digested with *Xba*I and *Pst*I, and cloned into the corresponding site of the binary vector pCAMBIA2300. The resulting pC2300-*OsChl P* plasmid, which contained the *OsChl P* sequence driven by the actin 1 promoter, was introduced into *Agrobacterium tumefaciens* strain EHA105, and transformed to the *502ys* mutant for complementation test. To identify whether the wild-type *OsChl P* gene were inserted into the mutant, the *502ys* mutant and its transgenic plants were amplified using primer 2 F (5′-GAATCCCTCAGCATTGTTC-3′) and primer 2R (5′-AGGGCAGCATAAATCAAGT-3′) that were designed to anneal to sequences located on the actin 1 promoter and *OsChl P* gene, respectively, in which the PCR conditions were: 94°C for 3 min; 30 cycles of 94°C for 30 s, 55°C for 30 s, 72°C for 1 min; and finally 72°C for 7 min.

## Results

### Characteristics of the *502ys* mutant

**Figure 1:**
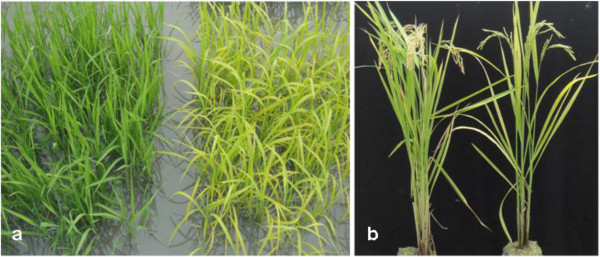
**Plant phenotype of the** ***502ys*** **mutant. a**. Four-week-old plants of wild-type Nipponbare (left) and *502ys* mutant (right). **b**. Plants of wild-type Nipponbare (left) and *502ys* mutant (right) at mature stage.

The *502ys* mutant was isolated from *japonica* rice cv Nipponbare via EMS mutagenesis. The mutant exhibited a yellow-green leaf phenotype throughout development and retarded growth rate (Figure 1). Although its days to heading increased 10.4 d, its height of plants and number of productive panicles decreased by 9.8% and 44.0% compared with the wild type, respectively (Table 1). Besides, its seed-setting rate and 1000-grain weight also declined by 9.1% and 6.2%, respectively.

**Table 1:** **Comparison of major agronomic traits between the** ***502ys*** **mutant and its wild-type Nipponbare**

Traits	Nipponbare (WT)	***502ys***	Compared with WT (%)
Days to heading (d)	87.3 ± 0.6	97.7 ± 2.5	+11.9%^*^
Plant height (cm)	104.0 ± 2.4	93.8 ± 1.6	−9.8%^*^
No. of productive panicles per plant	10.9 ± 0.7	6.1 ± 0.6	−44.0%^*^
No. of spikelets per panicle	116.3 ± 3.2	113.1 ± 2.4	−2.8%
Seed-setting rate (%)	87.4 ± 2.5	78.3 ± 1.9	−9.1%^*^
1000-grain weight (g)	27.4 ± 0.3	25.7 ± 0.4	−6.2%^*^

^*^Significantly different at *P* = 0.05.

To characterize the yellow-green phenotype of the *502ys* mutant, we measured its Chl contents, and found its contents of Chl *a*, *b*, and total Chls to be 28.4% to 72.8%, 17.6% to 50.7%, and 26.2% to 67.3% of those in wild type, respectively (Table 2), which indicated that the mutant phenotype resulted from reduced Chl level.

**Table 2:** **Pigment contents in leaves of the** ***502ys*** **mutant and its wild-type Nipponbare, in mg g fresh weight** ^**-1**^

Growth stage	Line	Total Chl	Chl***a*** ^a^	Chl***b*** ^a^	Chl a/b Ratio
Seedling stage	Wild-type	2.84 ± 0.19	2.13 ± 0.10	0.71 ± 0.09	3.00 ± 0.14
	*502ys*	1.91 ± 0.08	1.55 ± 0.09	0.36 ± 0.03	4.31 ± 0.32
Heading stage	Wild-type	3.36 ± 0.03	2.68 ± 0.02	0.68 ± 0.06	3.94 ± 0.22
	*502ys*	0.88 ± 0.04	0.76 ± 0.02	0.12 ± 0.02	6.33 ± 0.48

^a^Chls were measured in 80% acetone extracts from the first, second and third leaves from the top at indicated growth stages. Values shown are the mean ± SD from five independent determinations.

**Figure 2:**
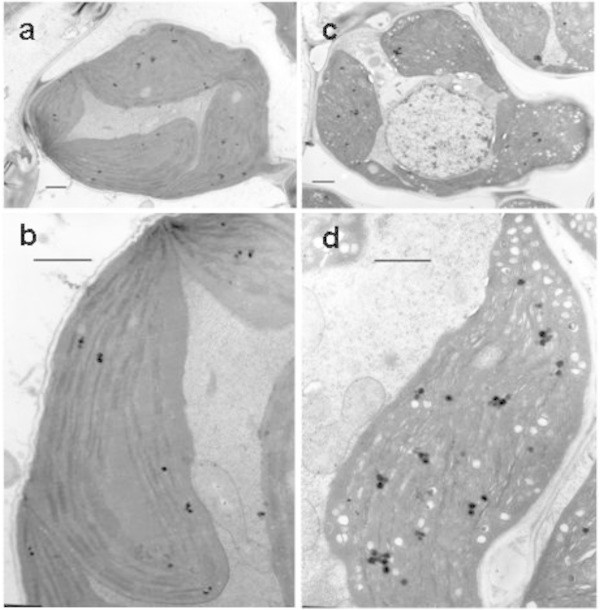
**Electron microscopic analysis of the rice** ***502ys*** **mutant. a**. and **b**. Mesophyll cells and chloroplast of wild-type Nipponbare, respectively. **c**. and **d**. Mesophyll cell and chloroplast of *502ys* mutant, respectively. Bar equals 1 μm.

To explore whether low Chl content in the mutant leaves affects the chloroplast development and morphology, we examined the ultrastructure of chloroplast using transmission electron microscopy. In wild type, the chloroplasts displayed well-developed membrane systems composed of grana connected by stroma lamellae (Figure 2a and b). However, the grana stacks in the *502ys* mutant appeared less dense compared with that in wild type. The thylakoid membrane systems of the mutant chloroplasts were disturbed, and the membrane spacing was not as clear as that in wild-type chloroplasts. In addition, the mutant chloroplasts exhibited vacuolation (Figure 2c and d). These results indicated that the development of chloroplast was suppressed in the *502ys* mutant.

### Map-based cloning of the mutant gene

The *502ys* mutant was crossed with normal green variety Minghui 63 and wild-type Nipponbare. As result, all F_1_ plants displayed normal green phenotype. F_2_ population from the two crosses showed a segregation ratio of 3:1 (green:yellow-green plants, χ^2^ < χ^2^_0.05_ = 3.84, P > 0.05). The data suggested that the yellow-green phenotype of *502ys* mutant is controlled by a single recessive nuclear gene.

**Figure 3:**
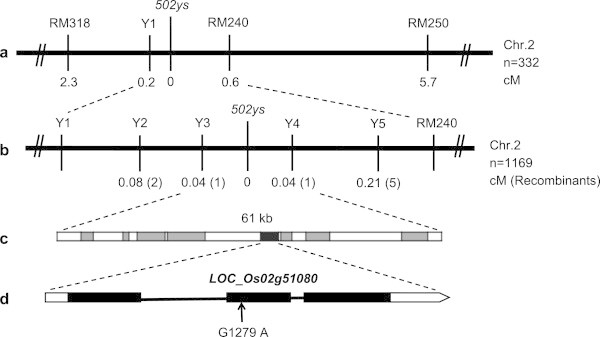
**Fine mapping of the** ***502ys*** **locus** ***.*** **a**l. The 5*02ys* locus was mapped to a region between InDel marker Y1 and SSR marker RM240 on the long arm of rice chromosome 2. **b**. The 5*02ys* locus was finely mapped to a region between InDel markers Y3 and Y4*.* **c**. The 61-kb region contains eight putative genes. **d**. The candidate *LOC_Os02g51080* gene is comprised of three exons and two introns, and a single nucleotide G-to-A substitution at position 1279 of its coding region was found in the *502ys* mutant.

To map the *502ys* locus, an F_2_ mapping population was generated from the cross between the *502ys* mutant with Minghui 63 (an *indica* cultivar). The *502ys* mutant gene was mapped to an interval between the SSR markers RM318 and RM240 on the long arm of chromosome 2 (Figure 3a). To further define the position of the *502ys* gene, six InDel makers were developed (Table 3). As result, the *502ys* locus was narrowed down to a 61-kb region between the two InDel makers Y3 and Y4 (Figure 3b).

**Table 3:** **Insertion/deletion (InDel) makers used for fine mapping of the** ***502ys*** **locus**

Maker	Forward primer (5′ ***–*** 3′)	Reverse primer (5′ ***–*** 3′)
Y1	GAAGATGATGCCAACTGA	GAGCCAATCCACAATACA
Y2	CCTCCTTAACTTTGCCTCTG	AGCCAGTAATAGCGTCCTCA
Y3	CCGTGGTTCAGTCAAAGA	AACGCGGTGATACATTCC
Y4	TATGGACGAGATGAGGAG	TAAGAGGCAGGTTGAAAA
Y5	AGAGGCGAGGAGGATTGG	AGGGCAGTAGCAGTGCAAGA

Within the Y3 and Y4 region, eight putative genes had been annotated in Rice Genome Annotation Project (http://rice.plantbiology.msu.edu). According to recombinants between the two InDel markers and the *502ys* mutant gene, we found one gene (*LOC_Os02g51080*) encoding a putative FAD binding domain containing protein, and sequence BLAST showed its encoded product has a significant similarity to CHL P (73% identities) in Arabidopsis. Then, we cloned the *LOC_Os02g51080* gene from the *502ys* mutant and its wild-type, respectively. DNA sequencing results revealed a single nucleotide G-to-A substitution at position 1279 of its coding region in *502ys* mutant. Next, its cDNA sequence was amplified by reverse transcription (RT)-PCR, and the G-to-A substitution at codon 616 (corresponding to position 1279 in the coding region) was confirmed in the mutant, which resulted in an amino acid change from Gly-206 to Ser (Figure 3d) in the encoded protein. Thus, *LOC_Os02g51080* gene was considered as the candidate gene of *502ys*, and designated tentatively as *OsChl P* gene. In addition, we also sequenced the 1975-bp upsteam of *LOC_Os02g51080* gene and found its promoter region did not change in the *502ys* mutant.

### Characterization of the *OsChl P* gene

Sequence comparison between genomic DNA and cDNA revealed that the *OsChl P* gene is comprised of three exons and two introns. The cDNA sequence of *OsChl P* consists of 1392 bp and encodes a 463-amino acid protein with a molecular mass of approximately 50 kD. OsCHL P protein contains an apparent chloroplast-targeting sequence of 37 amino acid residues at its N terminus (http://www.cbs.dtu.dk/services/TargetP/; Additional file 1: Figure S1).

**Figure 4:**
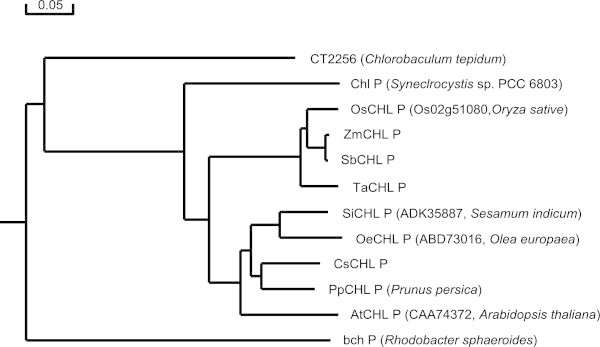
**Phylogenetic tree representing alignment of OsCHL P protein and its homologues.** The rooted neighbor-joining tree using percentage identities was constructed based on a multiple sequence alignment generated with the program DNAMAN. Accession numbers for the respective protein sequences are as follows: *Oryza sativa* (OsCHL P, LOC_Os02g51080), *Sorghum bicolor* (SbCHL P, XP_002454287), *Zea mays* (ZmCHL P, NP_001149382), *Triticum aestivum* (TaCHL P, AAZ67145), *Arabidopsis thaliana* (AtCHL P, CAA74372), *Cucumis sativus* (CsCHL P, XP_004138167), *Prunus persica* (PpCHL P, EMJ10306), *Sesamum indicum* (SiCHL P, ADK35887), *Olea europaea* (OeCHL P, ABD73016), *Synechocystis* sp. PCC 6803 (Chl P, NP_441659), *Chlorobium tepidum* TLS (CT2225, AAM73471), *Rhodobacter sphaeroides* (bch P, AAF24283). Scale represents percentage substitution per site.

BlastP search indicated that CHL P exists widely in various species, including higher plant, moss, Chlorophyceae and Cyanobacteria. Multiple amino acid sequence alignment suggested that OsCHL P has a high similarity to monocotyledonous plant sorghum (*Sorghum bicolor*), maize (*Zea mays*) and wheat (*Triticum aestivum*) CHL P, and dicotyledons plant cucumer (*Cucumis sativus*) and Arabidopsis CHL P, with identity of 93%, 92%, 91%, 81% and 73%, respectively. In addition, OsCHL P is also 77% and 76% identical to CHL P proteins in fruiter species, olive and peach, respectively (Additional file 1: Figure S1). At the same time, we analyzed the possible phylogenetic relationships between OsCHL P and its related proteins from higher plants and other photosynthetic organisms, indicating that the OsCHL P is more closely related to CHL P proteins from the monocotyledonous plants sorghum, maize and wheat than to those of other species (Figure 4).

### Analysis of chlorophylls

**Figure 5:**
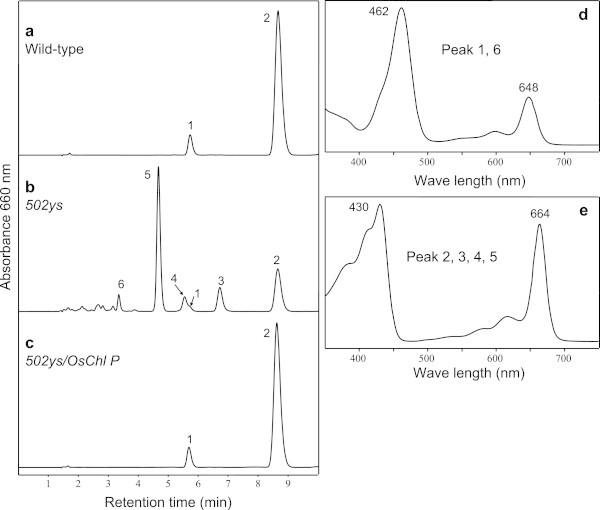
**HPLC analysis of Chls.** The elution profiles of wild type **(a)**, *502ys* mutant **(b)** and *502ys/OsCHL P* transgenic line **(c)** were detected at 660 nm. Peak 1, Chl *b*; peak 2, Chl *a*; peak 3, 4 and 5, Chl_THGG_ *a*, Chl_DHGG_ *a* and Chl_GG_ *a*, respectively; peak 6, Chl_GG_ *b*. The absorption spectra of the peaks 1 and 6 **(d)** and the peaks 2, 3, 4 and 5 **(e)** in acetone were compared, respectively.

Because CHL P catalyzes the reduction of Chl_GG_ to Chl_Phy_ (Keller et al. 1998), the final product of Chl biosynthesis should be Chl_GG_ when the plants have a defect in CHL P. So we examined Chl compositions of the *502ys* mutant by HPLC. The result showed that the majority of Chl molecules in the mutant were conjugated with an unsaturated geranylgeraniol side chain, in addition to small amount of normal Chl_Phy_ (Figure 5;Tanaka et al. 2010;Zhou et al. 2013), indicated that the mutant has a defective OsCHL P. Therefore, we concluded the candidate gene underlying the *502ys* mutant phenotype is *OsChl P*.

### Complementation analysis

**Figure 6:**
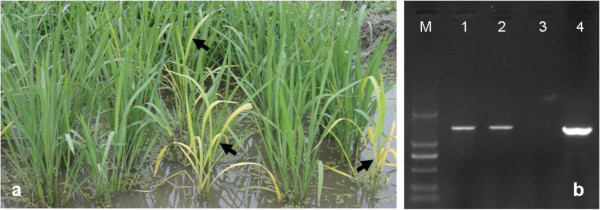
**Complementation of the** ***502ys*** **mutant by the wild-type** ***OsChl P*** **gene. a**. Phenotype of T_0_ transgenic plants. Arrowheads indicated PCR-negative transgenic plants, the others plants were PCR-positive transgenic plants. **b**. Identification of transgenic plants by PCR. M, DL-2000 marker; 1 and 2, PCR-positive transgenic plants; 3, The *502ys* mutant (PCR-negative control) or PCR-negative transgenic plants; 4, pC2300-*OsChl P* plasmid (control).

The identity of *OsChl P* was subsequently confirmed by genetic complementation experiments. The plasmid pC2300-*OsChl P*, containing the entire *OsChl P* (*LOC_Os02g51080*) genomic sequence of the wild-type Nipponbare under the control of the actin 1 promoter, was introduced into the *502ys* mutant, and 87 independent transgenic lines were obtained. Among them, 78 PCR-positive lines showed a complementation of the *502ys* phenotype and their growth rates were nearly identical to those of wild-type plants, whereas the other 9 PCR-negative lines failed to rescue the *502ys* mutant (Figure 6). Furthermore, HPLC analyses confirmed that the PCR-positive transgenic lines containing the wild-type *OsChl P* gene all accumulated normal Chl_phy_ (Figure 5C). Therefore, we concluded that we succeeded to clone *Chl P* gene in rice, and that the *502ys* mutant resulted from a single base pair mutation in *OsChl P* gene.

## Discussion

CHL P reduces GGPP to PhyPP and provides the side chain for Chl, tocopherols, and plastoquinones, which is prerequisite to their integration into plastid membranes (Soll et al. 1980,1983;Soll 1987;Bollivar et al. 1994;Graßes et al. 2001;Giannino et al. 2004). Addlesee et al. (1996) cloned the *chlP* gene from the cyanobacterium *Syneclrocysti*s sp. PCC 6803, and subsequently demonstrated this cyanobacterium gene was able to partially complement a *bchP* mutant of purple photosynthetic bacterium *R. sphaeroides*. Addlesee and Hunter (1999) identified the *bchP* gene in *Rhodobacter sphaeroides* transposon mutant, and demonstrated the activity of a *bchP* gene product using the recombinant protein. Keller et al. (1998) cloned a cDNA encoding CHL P from *Arabidopsis thaliana* and suggested that a multifunctional CHL P catalyzed the stepwise reduction of Chl_GG_*a* into Chl_phy_*a* as well as the reduction of GGPP into phyPP. Tanaka et al. (1999) cloned a tobacco cDNA sequence encoding CHL P, and showed reduction in Chl and tocopherol contents and accumulation of partially Chl_GG_ in transgenic tobacco expressing antisense *Chl P*. Giannino et al. (2004) identified a *Chl P* gene in peach and suggested that its expression was regulated by photosynthetic activity. Bruno et al. (2009) characterized a *Chl P* gene in olive, and found that *OeChl P* expression was modulated during leaf and fruit development and rapidly enhanced under stress conditions. Park et al. (2010) isolated a *Chl P* cDNA in sesame and showed *SiChl P* expression was induced by light but repressed by dark, ABA and ethylene. However, no *Chl P* gene had been identified in monocotyledonous plants until 2012. Most recently, Zhou et al. (2013) isolated a yellow leaf mutant (*lyl1*) from the progeny of a *japonica* rice ZH11 treated with ^60^Co radiation, and demonstrated the *lyl1* mutant phenotype was caused by a point mutation in the *LOC_Os02g51080* gene for CHL P via map-based cloning and RNA interference (RNAi) approaches. In present study, we isolated the yellow-green leaf mutant *502ys* from a *japonica* rice Nipponbare through EMS mutagenesis and cloned *OsChl P* gene (*LOC_Os02g51080*). In the *502ys* mutant, DNA sequence of the *OsChl P* gene had a single base pair mutation, resulting in an amino acid change in the encoded protein, and the majority of Chl molecules were conjugated with an unsaturated geranylgeraniol side chain. Furthermore, the mutant phenotype was rescued by transformation with the wild-type gene. Therefore, a *Chl P* gene was identified successfully in monocotyledonous plants.

Several Chl P (bchP) deficient mutants have been identified. A *bch P* mutant of the purple photosynthetic bacterium *Rhodobacter sphaeroides* accumulated only bChl_GG_ and the maximal photosynthetic growth rate were significantly reduced (Addlesee et al. 1996;Addlesee and Hunter 1999). Transgenic tobacco expressing antisense *Chl P* exhibited reduction in Chl and tocopherol contents, and up to 58% of Chls was esterified with geranylgeraniol instead of phytol under low light conditions (Tanaka et al. 1999). In addition, the transgenic plants were much more sensitive to light stress (Graßes et al. 2001). Graßes et al. (2001) suggested that Chl_GG_ did not affect the harvesting and transfer of light energy, but the reduced tocopherol content is a limiting factor for defensive reaction to photo-oxidative stress of the transgenic plants. In this study, the *502ys* mutant exhibited a yellow-green leaf phenotype, reduced Chl level, arrested chloroplast development, and retarded growth rate. HPLC analysis of Chls indicated that the *502ys* mutant accumulated Chls esterified with phytol, GG, dihydro-GG, and tetrahydro-GG. Our data are consistent with the above-mentioned reports that the *Chl P* mutants were capable of photosynthetic growth using Chls with an unsaturated geranylgeraniol side chain, although their growth rate was decreased remarkably.

Arabidopsis CHL P was able to catalyze the reduction of Chl_GG_*a* into Chl_Phy_*a* (Keller et al. 1998). The *502ys* mutant accumulated Chl_GG_*a*, Chl_DHGG_*a*, Chl_THGG_*a* and Chl_Phy_*a*, which implied that OsCHL P is also involved in the reduction of Chl_GG_*a*. However, when the recombinant OsCHL P protein was expressed in *E. coli*, we found that only a very small amounts of Chl_GG_*a* was detected to be converted to the Chl_DHGG_*a* after 5 h of incubation with the recombinant protein (data not show), implying its enzymatic activity were very low when Chl_GG_*a* was used as substrate. It is possible that the recombinant *OsChl P* product did not form a proper conformation, or the enzyme reaction system was not optimal, or additional proteins were required for its catalytic activity. In any case, further studies are necessary to clarify the function of the OsCHL P protein.

## Electronic supplementary material

Additional file 1: Figure S1: Alignment of the deduced amino acid sequence of OsCHL P and its homologues. Identical residues were boxed in black, similar residues (≥75% identical) were highlighted in gray. The red arrowhead indicates mutational site (G206S) of the *502ys* mutant, and the red underline indicates the putative chloroplast-targeting sequence of 37 amino acid residues at its N terminus. Accession numbers for the respective protein sequences are as Figure 4. (DOC 1 MB)

## Abbreviations

Chl: Chlorophyll
CHL P: Geranylgeranyl reductase
ChlPhy: Phytyl chlorophyll
ChlGG: Geranylgeranylated chlorophyll
ChlDHGG: Dihydrogeranylgeranyl chlorophyll
ChlTHGG: Tetrahydrogeranylgeranyl chlorophyll
GGPP: Geranylgeranyl diphosphate
PhyPP: Phytyl pyrophosphate.
