# Supplementary material for: Identification of a Geranylgeranyl reductase gene for chlorophyll synthesis in rice
Source: Springerplus. 2014 Apr 24;3:201. doi: 10.1186/2193-1801-3-201 (PMC4008729; doi:10.1186/2193-1801-3-201)
Supplement: Supplementary file 1 — Additional file 1: Figure S1: Alignment of the deduced amino acid sequence of OsCHL P and its homologues. Identical residues were boxed in black, similar residues (≥75% identical) were highlighted in gray. The red arrowhead indicates mutational site (G206S) of the 502ys mutant, and the red underline indicates the putative chloroplast-targeting sequence of 37 amino acid residues at its N terminus. Accession numbers for the respective protein sequences are as Figure 4. (DOC 1 MB) [file 40064_2014_920_MOESM1_ESM.doc]

**Additional file 1: Figure S1.** Alignment of the deduced amino acid sequence of OsCHL P and its homologues. Identical residues were boxed in black, similar residues (≥75% identical) were highlighted in gray. The red arrowhead indicates mutational site (G206S) of the *502ys* mutant, and the red underline indicates the putative chloroplast-targeting sequence of 37 amino acid residues at its N terminus. Accession numbers for the respective protein sequences are as Fig. 4.
